# Death from Chloroform

**Published:** 1854-01

**Authors:** John Roberton

**Affiliations:** Manchester.


### ARTICLE XIV.

*Death from Chloroform.* By John Roberton, Manchester.

Sir:—In common, I am sure, with many of your readers, I was shocked at the report, in your last number, of the death of a healthy young man under chloroform in the Edinburgh Infirmary. The impression is the more painful from what your reporter states, namely, that "a great error in the administration of chloroform prevails in that hospital;" that in hospital practice its administration is commonly entrusted to some raw dresser, who, thinking he cannot give enough of a good thing, squeezes a towel saturated with it over the mouth, where it is kept firmly applied, so that the admixture of chloroform with atmospheric air is rendered impossible.

Having been much in the habit of employing chloroform during the last five years, always with advantage, and without the occurrence of a single symptom to excite anxiety, it may not be unbecoming in me to make a few remarks on its administration.

Chloroform is a powerful agent, just as opium is a powerful agent. If improperly given, it will cause death; not, perhaps, with the same certainty as opium, for some constitutions resist its injurious influence in a wonderful manner; but still, as experience shows, it *may* produce death, which is all the more appalling from the suddenness, and from the impossibility, in nearly every case after the heart ceases to beat, of effecting resuscitation.

Now, in giving opium in the lying-in chamber in the form of laudanum, or Battley's sedative liquor, to allay false pains, or to soothe after an exhausting delivery, I am in the habit, as I suppose others are, of giving a certain number of drops, or of measuring, with care, a certain quantity. I don't think of giving the bottle to a nurse or other attendant, to pour out at random a dose for the patient to swallow. Were I to do so, I should hardly fail to have some ill effects. No one in his senses would administer, in this way, a preparation of opium.

Now, is not the remark equally applicable to the inhalation of chloroform? When administered in proper quantity, and there is a due admixture of atmospheric air, the effect is benign; but, when given in the *ad libitum* manner of some, what wonder if death or alarming consequences follow?

I will relate a case, on purpose to show the mode in which I

am accustomed to give chloroform, as also the rash manner in which it is sometimes employed by others.

On entering the chamber of a lady in labor, a few months ago, I found her provided with a bottle of Edinburgh chloroform. Her former labors having been hard, and the children of large size, she had determined, on this occasion, to have chloroform. When the stage for its employment arrived, I placed one end of a smooth napkin (so folded as to be about 7 inches in breadth by 10 in length) between her cheek and the pillow, she reclining on her side; and, requesting the husband's presence, I desired him to pour out a quantity not exceeding the fourth of a teaspoonful, which I sprinkled on the napkin opposite the patient's mouth and nose, at the same time bringing the other end over her face, yet not so as to approach it closely or to cover it; consequently, the air had free admission: three or four proportions of air to one of chloroform at least were inhaled. The sprinkling of a like quantity was repeated, about every three minutes, four times, when the patient was found to be asleep; and in a similar manner she was put to sleep five times in the course of three hours, at the end of which period labor terminated.

I have detailed this simple case for the following reason: I had noticed, at the outset, that the husband and the nurse smiled at the minute quantity I was administering; and, when sleep followed, I observed a marked expression of wonder, as if something quite unexpected had occurred. The explanation was this: The nurse had seen chloroform used once only before, and in large quantities, and she had been, I suppose, predicting that the lady's two-ounce bottle would be found too little, because, as she informed me, in the case referred to, the accoucheur had, from time to time, saturated a handkerchief with chloroform, and, during the labor, consumed nearly half a pint. The patient, she said, struggled and raved violently during its use, and yet without bad effects ensuing.

I have myself known chloroform administered in labor in dessert-spoonfuls until the pulse was at 40. Recently, too, I have known it, after being used cautiously for a short while with benign effect, given in the quantity of two drachms preparatory to the application of the forceps; and such were the alarming effects, that the patient turned herself suddenly on her back, and lay, with oustretched arms, for a few moments, pulseless.

Of course I do not mean that a drachm is not a safe, in many cases a proper, quantity, provided it be mixed with a reasonable proportion of atmospheric air, and is not repeated until the first quantity has been exhausted. But I do assert, that a larger quantity is dangerous, and I take the liberty emphatically to repeat, that it is as blameable in any surgeon to trifle with chloroform, by allowing it to be administered in random quantities by young and inexperienced hands, as it would be to trifle after the same manner (which, however, no surgeon does) with the preparations of opium.—*Medical Times and Gazette.*

